# A Spotlight on Preschool: The Influence of Family Factors on Children’s Early Literacy Skills

**DOI:** 10.1371/journal.pone.0095255

**Published:** 2014-04-21

**Authors:** Steve M. Heath, Dorothy V. M. Bishop, Kimberley E. Bloor, Gemma L. Boyle, Janet Fletcher, John H. Hogben, Charles A. Wigley, Stephanie H. M. Yeong

**Affiliations:** 1 School of Psychology, The University of Western Australia, Crawley, Australia; 2 Department of Experimental Psychology, The University of Oxford, Oxford, United Kingdom; Center for BrainHealth, University of Texas at Dallas, United States of America

## Abstract

**Rationale:**

Phonological awareness, letter knowledge, oral language (including sentence recall) and rapid automatised naming are acknowledged within-child predictors of literacy development. Separate research has identified family factors including socio-economic status, parents’ level of education and family history. However, both approaches have left unexplained significant amounts of variance in literacy outcomes. This longitudinal study sought to improve prospective classification accuracy for young children at risk of literacy failure by adding two new family measures (parents’ phonological awareness and parents’ perceived self-efficacy), and then combining the within-child and family factors.

**Method:**

Pre-literacy skills were measured in 102 four year olds (46 girls and 56 boys) at the beginning of Preschool, and then at the beginning and end of Kindergarten, when rapid automatised naming was also measured. Family factors data were collected at the beginning of Preschool, and children’s literacy outcomes were measured at the end of Year 1 (age 6–7 years).

**Results:**

Children from high-risk backgrounds showed poorer literacy outcomes than low-risk students, though three family factors (school socio-economic status, parents’ phonological awareness, and family history) typically accounted for less Year 1 variance than the within-child factors. Combining these family factors with the end of Kindergarten within-child factors provided the most accurate classification (i.e., sensitivity = .85; specificity = .90; overall correct = .88).

**Implications:**

Our approach would identify at-risk children for intervention before they began to fail. Moreover, it would be cost-effective because although few at-risk children would be missed, allocation of unnecessary educational resources would be minimised.

## Introduction

Many children fail to acquire literacy skills despite adequate intelligence and opportunity. Recent national literacy testing indicated that 25% of Australian Year 9 students achieved at or below the benchmark for minimum achievement in reading [1]. The identification of such children before they begin to struggle at school is a matter of significant concern to educators and policy makers as well as to parents. In Australia, the screening of children as they enter school at four and five years of age is becoming an increasingly common practice but for this process to be effective, it is essential that screening procedures are accurate and efficient. Understanding the factors that contribute to children’s early literacy skill development is of critical importance for researchers, policy makers, and educators [2]. While a large body of research has detailed several important early predictors of later reading achievements, many studies focus solely on within-child factors (e.g., phonological skills) or, alternatively, on environmental or genetic contributions, but not the interactive contribution of both child and family factors.

Within the child, there is a cluster of skills known to be fundamental to literacy development. These emergent literacy skills are phonological awareness (PA; i.e., an individual’s awareness of the sound structure of language), alphabet (i.e., letter) knowledge, and oral language, including sentence recall, vocabulary and grammatical understanding [3]–[10]. Children who enter school lacking these key skills are at increased risk of difficulties in literacy acquisition. These at-risk children rarely catch up to their peers [11], [12]. In addition to differences in skill levels across children, there is also great variation in the home environments from which children enter school, as well as in the characteristics and skills of their parents. Parent and home environment factors can have important implications for children’s literacy acquisition [13].

### Within-child Predictors of Later Literacy Skill

Although the direction of causality is not clear [14] it is well-established in the literature that a child’s levels of alphabetic knowledge and PA provide crucial information about their likelihood of literacy failure (or success). The capacity of a child to identify and work with speech sounds and the letters that map onto them in English has repeatedly been found to be an excellent predictor of later reading and spelling skill [6], [15], [16]. Children with strong alphabetic knowledge tend to go on to have strong literacy skills. Children’s oral language skill (including both receptive and expressive components) has also been identified as an important predictor of literacy development [17]. A further predictor of unique variance in literacy achievement is a child’s rapid automatized naming (RAN; usually operationally defined as how fast they can name highly-familiar items such as digits, letters, colours and objects) [18]. Research has indicated that this variable contributes independent variance to literacy outcomes and appears to relate particularly to the development of fluency and automaticity in literacy [6], [19].

### The Role of Families in Children’s Literacy Development - What can Parents Contribute?

Parents, as children’s first teachers [20] play a crucially important role in building their offspring’s emergent literacy knowledge and skills [21]. Consequently, an extensive body of research has documented the importance of a wide range of family and environmental factors in providing the context for children’s early literacy learning prior to and upon entering formal schooling.

#### Socio-economic status

Socio-economic status (SES), typically determined through family income, parent education level, and/or parent occupation, has frequently been used in studies of children’s early literacy achievement [22]–[24]. Children from varying SES levels have been found to differ significantly in the exposure they receive to print (e.g., parent-child reading), opportunities to engage in literacy-related activities, and the availability of literacy-related materials (e.g., books) in the home [25]–[30]. These differences may translate into early variations in the emergent literacy skills needed for reading. Children from low SES homes tend to perform less well on measures of print knowledge, exhibit delayed PA, and are at risk of developing reading difficulties [8], [22], [31]–[33]. Furthermore, studies indicate that children from socially and economically disadvantaged backgrounds have substantially smaller vocabularies than their more advantaged peers [34], [35], thus compromising the development of comprehension skills and PA [36], [37].

#### Mother’s level of education

While it is generally accepted that a parent’s level of education, particularly a mother’s, has an important relationship with children’s academic attainment in middle and late adolescence [38]–[40], findings concerning the relationship between mothers’ education and preschoolers’ emergent literacy skills have been mixed. Some studies report a strong relationship between maternal education and children’s literacy development [41], [42] while others have found no meaningful difference between children with mothers from either high or low educational backgrounds [43]. Christian, Morrison and Bryant [44] found that Kindergarten children of less educated mothers outperformed those of more highly educated mothers who engaged in fewer home literacy activities. This suggests that parental behaviours known to promote literacy development do not necessarily result from educational, social, and economic advantages.

#### Family history

It has long been recognised that reading difficulties aggregate in families [45]. Scarborough [46] reported on a number of studies which investigated the familial transmission of difficulties in reading and found that the incidence of reading disability in children of affected parents ranged from 23.0% to 62.0% (with an average of 38.5%). Substantial evidence indicates that children of parents and siblings with histories of reading disability are at greater risk of developing literacy problems [47]–[49]. The familial nature of reading disability is consistent with research indicating that a predisposition to literacy difficulties is highly heritable, and with family studies of reading disability that confirm a role for genetic transference [50]–[52]. However, as Olson and colleagues point out [53], the home environment and other contextual aspects also play an important role in addition to the contribution of genes. Moreover, the possible interaction between genetic and environmental factors is suggested by Pennington et al.’s [54] report that the effect of environment may differ for those at high versus low genetic risk of reading difficulties.

#### Parents’ phonological awareness

One of the key aspects of family risk status that has yet to be explored is the PA of parents. Considerable research shows that deficits in the acquisition of PA are a primary cause of reading disability [52], [55] and that adults with a childhood history of reading difficulties also exhibit poor PA [56]. Preschoolers who become disabled readers are characterised by weak emergent literacy skills, particularly PA, and infrequent exposure to books [57], [58]. While extensive research has investigated the familial incidence of reading disability, more recent evidence from a twin study by Byrne, Olson, Stefan, Wadsworth, Corley, DeFries and Willcutt [59] indicates significant heritability for a composite measure of PA in preschoolers without a family history of reading disability. Thus, the literature provides support for the possibility of a relationship between parents’ level of PA and preschoolers’ emergent literacy skills, particularly PA.

#### Parents’ perceived self-efficacy

Parents’ perceived self-efficacy (PSE) is another factor hypothesised to influence parents’ ability to support children’s learning. From Bandura’s [60] work on personal efficacy comes the notion that parents have efficacy beliefs relating to whether they can contribute to their child’s educational experiences, and if so how [61]. Parental self-efficacy has been shown to act as a mediating variable linking family characteristics and the home environment. Parents with higher levels of self-efficacy create more positive learning environments, spend more time engaging in educational activities, and tend to have higher levels of parent involvement [61], [62].

While research indicates that parental PSE is associated with parental competence and level of involvement [63], the relationship between parental self-efficacy beliefs and children’s educational outcomes has received little attention and further examination of these relationships is needed.

### Parental Factors as Predictors of Literacy Difficulties

There is evidence for an impact of these family factors on children’s literacy development, but there is little research on how well family factors can predict “at-risk” children. Studies that investigate the relationships between family factors and child literacy outcomes tend to focus separately on parental education, genetic contributions or socio-economic factors, For example, Leppänen, Aunola, Niemi and Nurmi [64] evaluated both child (PA, letter knowledge, and listening comprehension) and parent (mother’s level of education) factors but looked only at the individual contribution of these factors to children’s literacy skill at Grade 4. Other research [59] has examined the relationships between genetics, home environmental factors (e.g., family engagement in literacy activities) and child factors (PA, RAN and verbal memory), which have then been independently related to outcomes on reading and spelling measures [13]. However, the cumulative influence of heritability and other family factors (such as maternal education and socio-economics) has not been considered. Therefore, while much is known about within-child factors that are strongly related to literacy development (i.e., PA, letter knowledge and RAN), there are very few studies that combine these within-child predictors with family factors to improve the prediction of reading skill.

An exception is a study by Heath, Claessen, Fletcher, Hogben and Leitao [65] who combined information about family and child indicators and found that the best prediction of literacy difficulties in Year 2 students was gained by evaluating a child’s PA, letter knowledge, RAN, and short-term memory (as measured by repetition of sentences), in addition to mothers’ levels of education. Heath and colleagues suggested a screening tool whereby children who fell below the specified cut-offs on any three out of five of these within-child plus family factors in Kindergarten (five years of age) were at risk of developing literacy difficulties. This classification achieved a sensitivity of.86 for identifying children at last one standard deviation below the mean on a composite literacy measure at the end of Year 2, though there were concerns about less than optimal specificity (.69).

### Effective Identification of Children at Risk Before Formal Literacy Instruction

A critical issue in the detection of risk factors for literacy failure in young children is the age at which children can be accurately identified as being likely to struggle. Identification of children at risk prior to formal schooling and even on entry to Kindergarten (at five years of age), proves difficult. Some researchers suggest that Preschool (four years of age) PA cannot be reliably used as a predictor of literacy difficulties due to the instability of preschoolers’ performance on measures of PA at that time [66]. Consequently, most studies have focused on the identification of Kindergarten children at 4.5 to 6 years of age who are at risk of developing later literacy difficulties [67]. However, the earlier a child can be identified as “at-risk”, the earlier appropriate intervention can be provided. Also, as Byrne and colleagues [59] point out, the role that reading-related skills play in the development of literacy is likely to be much clearer if these are evaluated when the child’s exposure to formal literacy instruction is minimal.

There is support for measuring possible predictors earlier than the first year of school (Kindergarten, at 5 years of age). Schatschneider and colleagues [6] found that end of Kindergarten predictors held a stronger relationship with Grade 1 achievements in literacy than predictors measured at the beginning of Kindergarten. However, the differences between beginning and end of Kindergarten predictions were not large or statistically significant. Scarborough [67] reported that the size of correlations between reading and pre-literacy measures is similar in Kindergarten samples (five years of age) and pre-Kindergarten samples (three to five years of age). It has been suggested that the practical utility of identifying children at such an early stage may be limited [67]. However, in the Western Australian context, children can be readily assessed at the beginning of the Preschool year (four years of age) and early intervention is possible during this time. This is particularly important since researchers have emphasised the value of earlier identification of children who come from high-risk backgrounds where there is a family history of literacy-learning difficulties [67], [68].

### Present Research

While some studies have examined the familial component of literacy learning difficulties and others have explored the importance of a positive home environment for fostering developing literacy, there has been limited investigation of the *combined* effects of parent factors, including the mother’s level of education, whether a person within the family identifies as having a literacy learning problem, and the socio-economic status of the family.

This study examined SES, mother’s education and family risk status and their role in emergent literacy, and also introduced two new family factors: parents’ PA and PSE. The aim of this research was to examine the relative contributions of family and within-child factors, and the usefulness of both types of factors for prospective classification of children at risk for literacy failure. This longitudinal project tracked children from four years of age through to six years of age. It shifted the focus from predictors that can be identified in Kindergarten (when children are five years of age) to Preschool (when children are four years of age) as there has been limited examination of these factors in the younger age group.

It was hypothesised that:

- Children from backgrounds which might be expected to place them at high-risk (i.e., operationally defined here as their risk status on five factors suggested by the recent literature as influencing literacy development: low school SES, a family member with a literacy-learning difficulty, and low mother’s education, PA and PSE) would perform poorly on measures of pre- and early literacy when compared to children from low-risk backgrounds.
- A combination of child and family factors would provide better prediction of early literacy skills than within-child factors alone.
- Supplementing within-child factors with our additional family factors would offer more accurate prospective classification of children with respect to their early literacy development than has previously been attained with only a limited number of family factors included [65].

## Method

### Ethics Statement

The University of Western Australia Human Ethics Research Committee (RA/4/1/1970) approved the project. Individualised information packs were prepared for the school principals, teachers and parents approached to participate in this study. Written consent was obtained at each level with the parents/guardians of participating children also providing written consent for participation of their child. Participants (principals, teachers, parents/guardians or children) were free to withdraw at any time from the project without prejudice or the need to justify their decision. In the event of a withdrawal, all records related to their participation were destroyed unless an explicit or very strong implicit reason was given to retain the data (e.g., moving state but requesting to be informed about the study outcomes).

### Participants

An initial 162 families were recruited from schools across the Perth metropolitan area. These participants were part of a larger study, which evaluated a parent training program targeting skills for building resilience in children’s literacy. Fourteen families withdrew consent and had their data expunged and a further six provided no or minimal data. Of the remaining 142 families, 20 were removed from the analysis because of developmental issues known to have a significant impact on literacy development (e.g., autistic spectrum disorder, specific language impairment). Finally, 20 children did not complete all the literacy tasks and had their data withdrawn from this study. No significant differences were found between the families of these last children and the families of children included the study on the demographic data; gender: *χ* ^2^ (1) = .04, *ns*, school SES: *χ* ^2^ (3) = 3.28, *ns*, familial risk (e.g., having at least one first degree relative with literacy difficulty): *χ* ^2^ (1) = .33, *ns*, or on the initial child early literacy measures; reading readiness: *t*(115) = 1.35, *ns*, and early reading: *t*(114) = .39, *ns* (both at end of pre-primary; missing calculated pairwise). The final sample of children (*N* = 102; 46 females and 56 males) had a mean age of 51 months (*SD* = 3.62) at Time 1; 58 months at Time 2 (*SD* = 4.41); 69 months at Time 3 (*SD* = 3.75); and 80 months at Time 4 (*SD* = 3.78). Demographic characteristics of these children are shown in Table 1.

**Table 1:** Demographic characteristics of children in the study.

Variable group	Variable level	Frequency	Percentage
Mother’s education level	Completed Year 9 or lower	3	2.9
	Completed Year 10	14	13.7
	High school graduate and/or technical education diploma	21	20.6
	Technical education certificate	14	13.7
	University undergraduate degree	28	27.5
	University postgraduate degree	22	21.6
School SES	Low	18	17.6
	Middle	44	43.1
	High	40	39.2
Family risk	Yes	22	21.6
	No	80	78.4

### Materials

All measures used in this study, aside from the questionnaires developed specifically for the program, and the Sutherland Phonological Awareness Test – School Entry (SPAT-SE) [69], are well-recognised, standardised tests and the psychometric statistics provided below are sourced from the respective published manuals.

#### Child pre-literacy measures

Oral language ability was assessed with the Core Language subtests of the Clinical Evaluation of Language Fundamentals – Preschool - 2^nd^ Edition (CELF-PS-2) [70] in Preschool and Kindergarten; and with the Core Language subtests of the Clinical Evaluation of Language Fundamentals – Fourth Edition (CELF-4) [71] in Year 1. These subtests examine the receptive and expressive language abilities of children, such as whether they can understand spoken concepts and follow directions, and whether they can formulate sentences. Both versions of the CELF have been extensively tested and are considered gold standard tests by clinical practitioners. Reliability and validity data for both versions of the CELF are reported to be within the middle (*r*>.60) to high (*r>.*80*)* range across all subtests and composites, with most skewed toward the higher end.

Children’s PA was measured using the SPAT-SE [69] in Preschool and Kindergarten (i.e., Times 1, 2 and 3). At Time 3, three subtests from the Sutherland Phonological Awareness Test - Revised (SPAT-R) [72] were added (i.e., Final Phoneme Identification, Segmentation of CVC Words and Segmentation of Words with Blends) to prevent ceiling effects on the PA measure in the more phonologically sensitive Kindergarten children. The full SPAT-R was then administered at the end of Year 1 (i.e., Time 4). These two tests follow a developmental continuum that includes segmenting multisyllabic words into individual syllables, identifying rhyming words, initial and final phonemes, segmenting and deleting phonemes, and reading and spelling nonwords, with the SPAT-SE specifically designed for younger children and adapted from the SPAT-R. The SPAT-R has reported internal consistency (Cronbach’s Alpha) and inter-rater reliabilities of *r>*.95, and a concurrent validity based on the Woodcock Reading Mastery Test–Revised (WRMT-R) [73] Word Identification Subtest of *r = *.78. While reliability and validity statistics were not available for the version of the SPAT-SE used in this study, the test-retest correlations at Times 1 to 2 and Times 2 to 3 were both *r = .*62, and the average correlation between the SPAT-SE measures over Times 1 through 3 and the WMRT-R Word Identification Subtest at Time 4 was *r* = .49. In addition, internal consistency (Cronbach’s Alpha) for the five constituent SPAT-SE subtests at both Times 1 and 2 was *r = *.75, and at Time 3 with the added SPAT-R subtests it was *r = *.63.

Letter knowledge was assessed using the Phonological Abilities Test (PAT) [74] Letter Knowledge Subtest. Children were presented with lower-case letters printed on plain white cards and were asked to provide either the letter name or letter sound in Preschool and Kindergarten (i.e., Times 1, 2 and 3). In Year 1 (Time 4), for this study, children were asked to provide both the letter name and sound, and also the spoken sounds for four consonant blends and four digraphs. The test-retest reliability for the Letter Knowledge Subtest was *r* = .86 and the concurrent validly, compared to the British Abilities Scales (BAS) [75] test of Single Word Reading was *r = *.51. Sentence recall was measured using the Recalling Sentences Subtest from the CELF-PS-2 [70]. Children were presented aurally with sentences increasing in number of words and complexity and had to repeat the sentences verbatim.

RAN was measured at the end of Kindergarten using the Colours and Objects Subtests from the Comprehensive Test of Phonological Processing [18]. Children were asked to name as fast as they could a series of six colours or familiar objects repeated six times and arranged in random order. The completion times across two forms were summed for each subtest. Based on US data, Cronbach’s Alpha and test-retest reliablity were within the moderate to high range (*r = .*70−.92). Predictive validity for the Kindergarten CTOPP RAN colours/object composite based on subsequent WRMT-R Word Attack Subtest scores was *r* = .66 for Kindergarten to first grade and *r = *.70 for first to second grade.

#### Child early literacy measures

Word reading ability was assessed using the Word Identification Subtest (Form G) of the WRMT-R [76]. Children were required to read as many words as possible from a list that increased in difficulty. The split-half reliabilities are *r>*.90 for the Word Identification Subtest across all grades and concurrent validity for the Woodcock –Johnson Reading Tests [77] is *r = *.69 at Grade 3.

The Test of Word Reading Efficiency (TOWRE, Form A only) [78] was used to measure children’s word and nonword reading efficiency. Children were asked to read two separate lists of words and nonwords of increasing difficulty as fast as they could and the numbers of words and nonwords read accurately within 45 seconds were noted. Alternate-form reliablity coefficients across eight different sample populations (US data) were all above *r*>.95, with test-retest relabilities of *r*>.90 for the target age group. Concurrent validity for the TOWRE Sight Word Efficiency and Phonemic Decoding Efficiency scales with the WRMT-R Word Attack and Word Identification Subtests was cited as being *r*<.89 for Grade 1.

The Spelling Subtest from the Wechsler Individual Achievement Test –2^nd^ Edition: Australian Adaptation (WIAT-II) [79] was used as a measure of real word spelling ability. In addition, nonword spelling ability was measured using the Nonword Spelling Subtest of the Queensland University Inventory of Literacy (QUIL) [80]. In both tasks, single words and nonwords of increasing difficulty were presented orally to children, who were required to correctly spell real words and provide a plausible spelling for each nonword. The split-half reliability coefficients for the Spelling Subtest for the age range of interest (5–6 years) was reported as *r*>.92 and concurrent validity with the Wide Range Achievement Test – Third Edition [81], Spelling Subtest was *r = .*78. The QUIL test manual does not provide Cronbach Alpha’s or split-half reliability statistics for the Nonword Spelling Subtest because of the subjective nature of scoring but it does provide an inter-rater reliability estimate between two practitioners of 94%. The test manual also indicates concurrent validity of the Nonword Spelling Subtest by showing a significant correlation (*p*<.001) between it and the Modified Schonell Graded Spelling Test [82] though it does not provide a point estimate. However, our data show a QUIL Nonword Spelling and SPAT-R Nonword Spelling Subtest correlation of *r = *.76, suggesting adequate concurrent validity.

#### Family measures

A demographic questionnaire was developed to obtain information about both parents’ education level and family history of literacy difficulties. Positive familial risk for literacy difficulties was operationally defined as reporting at least one first degree relative (i.e., parent or full sibling) with a language/literacy problem. In addition, parents were administered another questionnaire which measured their sense of self-efficacy in supporting their child’s literacy and socio-emotional development. The 11 items were developed based on research on parents’ perceptions of parenting, coping and resilience [61], [83]. Parents were asked to rate how they felt about each statement on a five-point Likert scale. Items were piloted with a separate group of parents of Preschool children before being used in this study. Table S3 reports the factor structure of the questionnaire based on the pilot data. Based on the current sample population, Cronbach’s Alpha for all eleven items on this scale was *r = *.86 and there was a significant correlation (*r* = .27, *p*<.01) between the scale and concurrent parent ratings on total scores for the Strength and Difficulties Questionnaire (SDQ) [84].

A measure of SES for each family was obtained based on the socio-economic level of each school (school SES), as determined by the Western Australian Department of Education Socio-Economic Index (WADoE SEI). The socio-economic values are determined from the address of every student in the school, with the value calculated using data from the Australia Bureau of Statistics (ABS) for collection districts in which the addresses fall. The ABS values used in the calculation are education, occupation, aboriginality, income, and single parent status for the collection district.

Parents’ PA was assessed using the Nonword Segmentation and Phoneme Reversal Subtests from the CTOPP. These two supplementary subtests were chosen as they tap meta-analytic phoneme awareness, which has been more strongly linked to literacy in older individuals [66] at a difficulty level more suited to adults than the core subtests. For the Nonword Segmentation Subtest, parents were required to segment each nonword into its respective phoneme components, whereas for the Phoneme Reversal task, parents had to reverse the order of phonemes in aurally presented nonwords. The test-retest reliabilities for adults on the Nonword Segmentation and Phoneme Reversal Subtests are *r = *.72 and *r = *.81 respectively and the criterion validities for these subtests compared to the TOWRE Total Word Efficiency composite were *r* = .43 and *r* = .57.

### Procedure

Children in the final sample were assessed at four time points over the three year course of the study: beginning of Preschool (Time 1), beginning of Kindergarten (Time 2), end of Kindergarten (Time 3), and end of Year 1 (Time 4). Four pre-literacy skills (i.e., oral language, PA, letter knowledge, and sentence recall) were assessed at each time point, whereas RAN was only assessed at Time 3. With regard to the early literacy tasks, word reading was assessed at Times 3 and 4 but reading efficiency, real word and nonword spelling were only assessed at Time 4. All parent measures were administered at Time 1.

Participants were tested individually at the child’s school. At Time 1, children completed two sessions of approximately 20 minutes, and parents completed one session of approximately 15 minutes. At all other time points, children were assessed over two sessions of approximately 25 to 30 minutes in length. Whenever standardised tests were used, these were administered and scored according to the test instructions.

## Results

Where standard or scaled scores were used, these were derived from normative data in the relevant test manuals with the exception of parents’ PA. The CTOPP manual only provides data up to age 24 years 11 months, but this level was deemed to equate to adult performance, so parents 25 years or over were also scored on these norms. Missing scores on the pre-literacy tasks measured at T1 to T3 were replaced using means according to group membership based on the number of familial risk factors (see below). A Year 1 (T4) literacy composite for each child was derived by averaging the standard scores obtained on the single word reading and reading efficiency tasks, and the real and nonsense word spelling tasks. Correlations between all the early literacy tests are presented in Table S1.

The analyses reported below were conducted using the SPSS19 statistical package. However, due to concerns that the structure of some data sets threatened the usual assumptions for the analyses undertaken, results were validated using robust techniques outlined by Wilcox [85]. In each case, parallel tests were run with the requirement that at least two out of three robust test results confirmed the pattern revealed by the initial analysis before accepting that outcome. This benchmark was attained in all but two cases which are noted below.

### Pre- and Early Literacy Skills of High- and Low-risk Children based on Potential Familial Risk Factors

To explore the first hypothesis that children experiencing higher risk environments would have poorer outcomes on variables associated with pre- and early literacy skills, a number of ways of representing risk level and analytical methods were trialled. It was determined that the most parsimonious method was to develop a single categorical variable representing an overall level of hypothesized risk based on the five environmental factors. A median split was used to divide children into high- and low-risk groups on four of the five family factors (i.e., school SES, mother’s level of education, parents’ PA and parents’ PSE). On the fifth family risk factor, family history, a child was classified as being at high-risk if he or she had one or more immediate family members with a language/literacy problem. The number of familial risk factors (range: 0–5) for each child was summed. Examination of children’s performance on the literacy composite (i.e., after a year of formal literacy education) as a function of familial risk confirmed that children with more familial risk factors performed more poorly on the literacy composite; *F*(4, 97) = 7.05, *p<*.001. Pairwise comparisons (using bootstrap *n* = 1000) revealed that those children having zero to two risk factors performed significantly better on the literacy composite than those with three risk factors (0 risk factors: 95% CI [−21.90, −4.71], *p<.*01; 1 risk factor: 95% CI [−18.86 to −6.16], *p = .*001, and for 2 risk factors: 95% CI[−15.15, −2.17], *p<.*05,) or four risk factors (0 risk factors: 95% CI [−26.33 to −8.61], *p = .*001; 1 risk factor: 95% CI[−23.08, −10.00], *p = .*001, and for 2 risk factors: 95% CI [−19.59, −5.78], *p<.*01).

**Figure 1:**
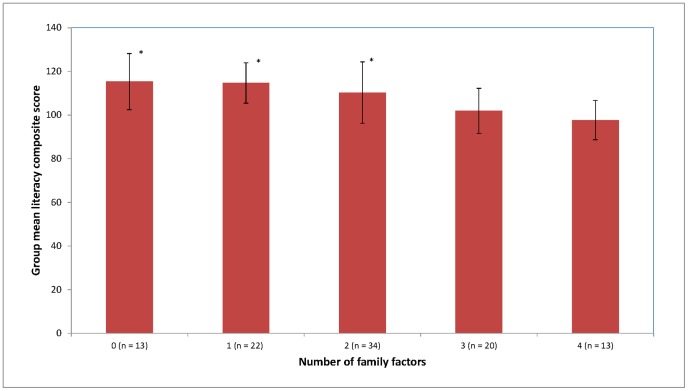
Literacy composite scores as a function of children’s number of potential familial risk factors. Risk factors were operationally defined here as: school SES, mother’s level of education, parents’ PA, parents’ PSE and family history of reading difficulty. Error bars denote standard deviations. *signifiactly different (*p*<.05) to those with 3 or 4 family factors. *NB:* No child was found to have all 5 familial risk factors.

Based on these results, and the fact that the largest decrease in performance was between children with two and three external risk factors (Figure 1), children with two or fewer factors were categorised as at low-risk, and children with three or more risk factors were categorised as at high-risk. This approach afforded a “snap shot” of the how children’s actual progress on well-known literacy and related measures at each time point differed according to their risk status. The results reported below using this approach to grouping were checked and found to be consistent with the various trial analyses we conducted using more discriminating methods of representing risk, (e.g., grouping based on the sum of risk factors; see Figure 1, or using quartile divisions rather than median splits). Additionally, this method limited the number of individual tests undertaken, thus limiting overall Type I error probabilities, while retaining a good level of useful descriptive information. Correlations are presented in Table S2 for the specific relationships between each original family factor variable and the literacy-related measures at Times 1, 2 and 3, and the literacy composite at Time 4. This affords the interested reader some insight into the associations between the continuous variables.

#### Pre-literacy skills

Tables 2a and 2b show the means and standard deviations of the pre-literacy tasks as a function of time for the low-risk and high-risk groups respectively. Separate 2 (risk: high vs. low) x 3 (time: T1 vs. T2 vs. T3) mixed ANOVAs were carried out for four pre-literacy skills (i.e., oral language, PA, letter knowledge, and sentence recall). There was a significant interaction between risk group and time for letter knowledge, *F*(2,200) = 6.84, *p* = .001, *η* ^2^_partial_ *= *.064. Further analyses to disambiguate this interaction showed that the low-risk group had better letter knowledge than the high-risk group at T1 and T2, but not at T3, indicating a significant improvement for that group from T2 to T3.

**Table 2:** *a.* Mean performance (SD) on the pre-literacy tasks for the low-risk (N = 69) group as a function of time; *b.* Mean performance (SD) on the pre-literacy tasks for the high-risk group (N = 33) as a function of time.

	Time	Oral language	PA	Letter knowledge	Sentence recall	Rapid Automatised Naming
a	T1	107.95 (8.57)	30.15 (9.48)	8.69 (7.31)	11.70 (2.05)	–
	T2	108.46 12.05)	37.02 (8.88)	14.25 (8.49)	11.14 (2.16)	–
	T3	108.59 10.31)	48.19 (7.37)	21.00 (4.61)	11.33 (1.99)	97.74 (15.73)
	T4	101.64 11.49)	67.58 25.38)	54.47 (5.31)	11.09 (2.33)	–
b	T1	103.60 10.23)	23.65 (9.25)	4.40 (5.53)	10.56 (2.46)	–
	T2	102.36 11.65)	30.70 (9.50)	8.39 (6.84)	9.55 (2.76)	–
	T3	100.45 11.11)	42.97 (8.03)	19.91 (4.68)	9.82 (2.46)	95.64 (13.65)
	T4	95.45 (13.68)	41.79 (21.63)	50.67 (6.54)	9.30 (3.09)	–

*Note:* Standard scores on measures of letter knowledge and PA were not available. Possible maximum score for Letter Knowledge at T1,T2 & T3 = 26, but at T4 = 60; possible maximum score for PA at T1, T2, & T3 = 50, but at T4 = 62 (see text). Scores on the Oral Language and RAN were standard scores (i.e., *M* = 100; *SD* = 15), and scores on the sentence recall were scaled scores (i.e., *M* = 10; *SD* = 3).

In addition, there were significant main effects of risk group, suggesting that children in the low-risk group performed significantly better than children in the high-risk group on each of the four pre-literacy skills (oral language: *F*(1,100) = 10.54, *p* = .002, *η* ^2^_partial_ *= *.095; PA: *F*(1,100) = 15.67, *p*<.001, *η* ^2^_partial_ *= *.136; letter knowledge: *F*(1,100) = 10.27, *p* = .002, *η* ^2^_partial_ *= *.093; sentence recall: *F*(1,100) = 12.31, *p* = .001, *η* ^2^_partial_ *= *.110) across all time points.

There were also significant main effects of time, with children performing significantly better at each time point on letter knowledge, *F*(2,200) = 226.96, *p*<.001, *η* ^2^_partial_ *= *.694, and PA, F(2,200) = 72.65, *p*<.001, *η* ^2^_partial_ *= *.421. The primary analysis also suggested that the children were significantly better on the sentence recall task, *F*(2,200) = 7.11, *p* = .001, *η* ^2^_partial_ *= *.066, at T1 compared to the other two time points. However, all three of the robust tests failed to confirm a significant trend suggesting the latter finding may be the result of an artefact within the data.

When these four pre-literacy skills were assessed in Year 1, a series of between-group ANOVAs showed significant differences between the high- and low-risk groups on oral language, *F*(1,100) = 5.70, *p* = .019, *η* ^2^_partial_ *= *.054, PA, *F*(1,100) = 22.65, *p*<.001, *η* ^2^_partial_ *= *.184, letter knowledge, *F*(1,100) = 9.89, *p* = .002, *η* ^2^_partial_ *= *.090, and sentence recall, *F*(1,100) = 10.57, *p* = .002, *η* ^2^_partial_ *= *.096.

As RAN was only assessed at T3, a between-group ANOVA was carried out to compare the performances of the high- and low-risk groups, but significant group differences were not found on this task (*p* = .512).

#### Early literacy skills

Table 3 shows the means and standard deviations of children’s performance on the early literacy tasks (i.e., reading and spelling skills at the end of Year 1). It is notable that the low-risk group appears to substantially outperform the normative sample on the measure of reading efficiency; however, this pattern is consistent with previous research findings for Western Australian children of this age (Heath et al., 2007). A 2 (risk: high- vs. low-risk) x 2 (time: T3 vs. T4) mixed ANOVA was conducted to investigate group differences in single word reading ability. Results showed that the children performed significantly better at T4 than at T3 on word reading, *F*(1,100) = 541.09, *p*<.001, *η* ^2^_partial_ *= *.844. A significant interaction between risk group and time was also found, *F*(1,100) = 17.89, *p*<.001, *η* ^2^_partial_ *= *.152. This was a reflection of children in the low-risk group showing a greater improvement in real word reading than the children in the high-risk group from T3, *F*(1,100) = 10.40, *p* = .002, *η* ^2^_partial_ *= *.094, to T4, *F*(1,100) = 31.30, *p*<.001, *η* ^2^_partial_ *= *.238. In addition, group differences were also observed in one-way ANOVAs conducted for the reading efficiency, single word and nonword spelling tasks, which were only assessed at T4. The children in the low-risk group had better reading efficiency, *F*(1,100) = 17.82, *p*<.001, *η* ^2^_partial_ *= *.151, and could spell more real words, *F*(1,100) = 15.91, *p*<.001, *η* ^2^_partial_ *= *.137, and nonwords, *F*(1,100) = 18.54, *p*<.001, *η* ^2^_partial_ *= *.156, than those in the high-risk group. It could be argued that the greater improvement in the low-risk group is an artefact of floor scores in the high-risk group, whereas if more simple words had been included, perhaps greater improvement could have been measured in the latter group. However, the groups differed greatly as to proportion of early non-readers, i.e., *p*(High-risk) = .56 and *p*(Low-risk) = .10, suggesting that this is unlikely to have made a significant difference. Moreover, even if improvement has occurred in some students that we have failed to capture in our analyses, it is quite obvious that on average, the high-risk group still lags far behind their low-risk peers on key literacy measures at the end of their first year of formal literacy education.

**Table 3:** Mean performance (SD) on the early literacy tasks as a function of risk group and time.

Risk group	T3 Word reading (/106)	T4 Word reading (/106)	T4 Reading efficiency	T4 Real word spelling (/53)	T4 Nonword spelling (/5)
Low-risk (*N* = 69)	11.22 (14.82)	43.7 (16.87)	112.06 (15.81)	16.48 (4.96)	2.09 (1.20)
High-risk (*N* = 33)	2.76 (3.64)	25.24 (12.43)	99.15 (11.02)	12.45 (4.32)	1.03 (1.08)

*Note:* All scores used were raw scores except for reading efficiency (standard scores, i.e., *M* = 100; *SD* = 15).

In summary, the results above support our hypothesis that children with more family risk factors (three or more) had poorer pre-literacy skills and eventually, poorer early literacy outcomes sustained over their first year of formal schooling, than children with two or fewer risk factors. Hence, family risk factors do influence children’s proficiency in pre-literacy skills and subsequent literacy outcomes.

### Predicting Early Literacy Skills

Separate regression analyses were carried out to determine which family risk factors, and child risk factors, measured at T1(Preschool) were important in predicting the children’s early literacy skills at T4 (Year 1) using the derived literacy composite (see above). First, we report the results of entering all predictors into the regression analyses, and then those of a stepwise regression of all predictors which helped us clarify whether or not these results were similar to the earlier analyses (see Table 4). Finally, hierarchical regressions were conducted to determine whether a combination of child and family risk factors provides better prediction of early literacy skills than child risk factors alone (see Tables 5a, 5b, and 5c).

**Table 4:** Family risk factors and child risk factors at T1, T2 and T3 that were significant (stepwise regressions for each factor set) in predicting early Year 1 literacy outcomes.

Factors	Significant variables	*β*	*t*	Adj. *R^2^*
Family risk factors	Family history	−.23	−2.38*	.20
	School SES	.25	2.75**	
	Parent PA	.26	2.69**	
T1 child risk factors	Letter knowledge	.45	5.31***	.29
	Sentence recall	.25	2.93**	
T2 child risk factors	Letter knowledge	.47	5.58***	.48
	Sentence recall	.23	2.91**	
	PA	.21	2.40*	
T3 child risk factors	Letter knowledge	.20	2.36*	.44
	PA	.26	2.99**	
	Oral language	.26	3.12*	
	RAN	.26	3.17**	

*p<.05.

**p<.01.

***p<.001.

**Table 5:** *a.* Hierarchical regression predicting early literacy outcomes from family and child risk factors at T1; *b.* Hierarchical regression predicting early literacy outcomes from family and child risk factors at T2; *c.* Hierarchical regression predicting early literacy outcomes from family and child risk factors at T3.

	Regression entry	Variable	*β*	*t*	Adj. *R* ^2^	Δ *R* ^2^
a	Step 1	Letter knowledge	.41	4.98***	.29	.30***
		Sentence recall	.19	2.38*		
	Step 2	School SES	.16	1.99*	.40	.13***
		Family history	−.11	−1.27		
		Parents’ PA	.27	3.23**		
b	Step 1	Letter knowledge	.46	5.53***	.48	.49***
		Sentence recall	.18	2.38*		
		PA	.14			
	Step 2	School SES	.14	1.94	.52	.13***
		Family history	−.08	−.99		
		Parents’ PA	.18	2.27*		
c	Step 1	Letter knowledge	.21	2.70**	.44	.46***
		PA	.24	3.09**		
		Oral language	.11	1.33		
		RAN	.33	4.50***		
	Step 2	School SES	.29	4.27***	.57	.14***
		Family history	−.14	−1.91		
		Parents’ PA	.19	2.68**		

*p<.05.

**p<.01.

***p<.001.

When school SES, mother’s level of education, parents’ PA, parents’ PSE, and family history were entered together, these factors explained a significant 23% of the variance in the literacy composite variable, *F*(5, 96) = 5.70, *p*<.001. However, only the beta values for school SES (*p* = .018), family history (*p* = .036) and parents’ PA (*p* = .013) were significant. A stepwise regression confirmed these three variables as important family factors in predicting early literacy in children, *F*(3, 98) = 9.31, *p*<.001, while mother’s level of education and parents’ PSE were not found to be significant predictors.

Oral language, letter knowledge, PA and sentence recall measured at T1 accounted for 31% of the literacy composite, *F*(4, 97) = 10.99, *p*<.001. However, the only significant predictor found was letter knowledge (*p*<.001). Results from a stepwise regression with all 4 predictors showed two predictors, letter knowledge and sentence recall, to be significant, *F*(2, 99) = 21.67, *p*<.001.

Though the contribution of family risk factors to Year 1 early literacy skills is less compared to child risk factors measured at the beginning of Preschool, a combination of both family and child risk factors does provide a better prediction of such skills than within-child factors alone (see Table 5). Further analyses using T2 and T3 predictors revealed similar overall patterns. However, although children’s letter knowledge was the strongest and most constant predictor, not all variables were consistently significant in predicting Year 1 literacy. In particular, children’s PA, which was not significant at T1, became important from T2 onwards.

### Prospective Classification of Children based on Family and Child Risk Factors

To determine how accurate it is to prospectively classify children into those who will perform below and above average in early literacy tasks using family and child risk factors, separate logistic regression analyses were conducted for each time point using a combination of family and child risk factors. Year 1 children were divided into the two groups by taking those more than.25 *SD* below the sample mean (*M* = 108.72, *SD* = 13.04) on the literacy composite score as those performing below average (*n* = 40). This cut-off was chosen to increase the chances of accurately identifying children who had already begun to lag behind in literacy (i.e., in time for prophylactic intervention). The initial model used child risk factors measured at Preschool and Kindergarten that significantly predicted the Year 1 literacy composite. Next, three factors, school SES, family history and parents’ PA, which were significant predictors of children’s early literacy scores, were added to determine if accuracy in identifying children would be enhanced by these additional familial risk factors.

#### Using Preschool (4 year olds) child risk factors and family risk factors

The initial model, which included T1 child factors (letter knowledge and sentence recall), was significant and able to distinguish children performing above and below average on the literacy composite score at Year 1 (*χ* ^2^(2)_ = _18.96, *p*<.001). These factors had an overall prediction success of 65.7% (77.4% for above average and 47.5% for below average). Both letter knowledge (*p* = .002) and sentence recall (*p* = .029) were significant contributors to the prediction. The addition of the three family factors to the model was significant (*χ* ^2^(3) = 16.13, *p* = .001) and this final model was a better fit to the data (*χ* ^2^(5) = 35.09, *p*<.001) than the initial model. The combination of T1 child factors and the three family factors had an overall prediction success of 77.5% (87.1% for above average and 62.5% for below average). This time, only letter knowledge was a significant contributor to the prediction among the child risk factors (*p* = .002), and parents’ PA (*p* = .001) was the only significant predictor among the family risk factors.

#### Using beginning Kindergarten (5 year olds) child risk factors and family risk factors

Child risk predictors measured at T2 (letter knowledge, PA, and sentence recall) were significant in classifying children performing above and below average on the literacy composite score at Year 1 (*χ* ^2^(3) = 44.20, *p*<.001). An overall prediction success of 82.4% was obtained (85.5% for above average and 77.5% for below average). PA (*p* = .018), letter knowledge (*p* = .006) and sentence recall (*p* = .003) contributed significantly to the prediction success. The addition of the three family risk factors did not add any significant accuracy to prediction success (*χ* ^2^(3)_ = _6.95, *p* = .073), though the final model remained significant (*χ* ^2^(6) = 51.15, *p*<.001). Two child risk factors (letter knowledge: *p* = .003 and sentence recall: *p* = .017) and parents’ PA were significant contributors (*p* = .018) in the final model.

#### Using end Kindergarten (5 year olds) child risk factors and family risk factors

An initial model with significant child risk predictors measured at T3 (letter knowledge, PA, oral language and RAN) was significant and reliably distinguished children who were performing above and below average on the literacy composite score in Year 1 (*χ* ^2^(4) = 46.61, *p*<.001). The overall classification accuracy using these factors was 80.4% (88.7% for above average and 67.5% for below average). PA (*p* = .009), letter knowledge (*p* = .042) and oral language (*p* = .004) contributed significantly to prediction of this model, but not RAN (*p* = .111). Additional family risk factors (*χ* ^2^(3) = 17.53, *p* = .001) added significantly to the accuracy of the final model (*χ* ^2^(7) = 64.13, *p*<.001). The combination of T3 child risk factors and family risk factors gave an overall classification accuracy of 88.2% (90.3% for above average and 85.0% for below average). The four child risk factors, PA (*p* = .020), letter knowledge (*p* = .007), oral language (*p* = .049) and RAN (*p* = .046) were significant child factors contributing to the model, whereas school SES (*p* = .014) and parents’ PA (*p* = .003) were significant among the family risk factors.

In summary, our results (see Table 6) showed that classification was most accurate at the end of Kindergarten with a combination of both child and family risk factors. The overall classification was 88.2% accurate and had high levels on specificity (90.3%) and sensitivity (85.0%). The addition of family risk factors at Preschool and at the end of Kindergarten, but not at the beginning of Kindergarten, provided better classification of children who would perform above and below average in literacy tasks at the end of Year1.

**Table 6:** Classification accuracy using within-child and family risk factors.

Time	Factors	Sensitivity	Specificity	Overall % accuracy
T1	Within-child factors	47.5%	77.4%	65.7%
	Additional family risk factors	62.5%	87.1%	77.5%
T2	Within-child factors	77.5%	85.5%	82.4%
	Additional family risk factors	75.0%	83.9%	80.4%
T3	Within-child factors	67.5%	88.7%	80.4%
	Additional family risk factors	85.0%	90.3%	88.2%

*Note.* Within-child factors at T1: letter knowledge & sentence recall; T2: letter knowledge, sentence recall & PA; T3: letter knowledge, PA, oral language & RAN. Additional family risk factors were: school SES, family history & parents’ PA.

## Discussion

Familial factors and within-child factors have been shown independently to be important for children’s early literacy skills, though hitherto little was known about the combined influence of these factors on children’s literacy skills. The present longitudinal study examined the effects of familial and child risk factors in children from four to six years of age by firstly, comparing children at high and low risk based on familial risk factors on their pre-literacy and early literacy skills; secondly, by establishing the combination of child and familial risk factors that would be most useful in predicting literacy skills; and finally, determining if a combination of child and familial factors would identify children likely to perform poorly in early literacy tasks more accurately.

### Influence of Familial Factors

Our results showed that children from backgrounds that placed them at high risk did perform more poorly on measures of pre-literacy (i.e., oral language, PA, letter knowledge and sentence recall) than children from low-risk backgrounds. In particular, having more family risk factors (three or more) could lead to weaker pre- and early literacy skills than having fewer or none. Though both high- and low-risk children improved in their performance on pre-literacy tasks over time, group differences persisted from Preschool to Year 1 and eventually, did result in poorer Year 1 literacy outcomes for the high-risk children. This suggests that high-risk children may not reach proficiency levels similar to those of low-risk children, even after a year of formal schooling. These high-risk children showed weaknesses in a broad range of pre-literacy abilities and will need a wider range of instructional support and interventions [23]. It is also likely that those children who are lagging behind may not catch up with other children, even after years of literacy education [86]. However, no group difference was found in RAN. This task measures how quickly children can gain access to verbal information stored in long-term memory and is related to fluency and automaticity in literacy. Hence, this may imply that familial conditions and environment do not influence naming skill in the same way as they appear to impact on other pre-literacy skills. This result is consistent with continued findings that RAN contributes unique variance to literacy (i.e., different from PA) and therefore, is dissociable from other pre-literacy skills [87].

### Combining Familial and Child Factors to Predict Literacy Skills

Next, a combination of family factors was found to significantly predict Year 1 literacy skills. Specifically, having low school SES, a family history of language/literacy difficulty, and low PA in parents could lead to poor literacy outcomes in children. The first two of these familial factors have been consistently found in the literature to independently relate to child outcomes. SES influences children’s opportunities for print exposure and engagement in literacy-related activities in the home, thus affecting emergent literacy skills [25] and children from low socio-economic backgrounds perform poorly on PA, and are at risk for literacy difficulties [31]–[33]. Other studies have shown that having an immediate family member with literacy difficulties can put one at risk of similar difficulties, suggesting a genetic contribution to the predisposition to developing poorer literacy skills [50], [51]. Less is known about the relationship between parents’ PA and children’s literacy abilities. We found that parents’ PA is a significant predictor of children’s early literacy skills: this could reflect shared genetic influences on PA in both parent and child [59], but might indicate cultural transmission, if parents with low PA have difficulties providing support for their children’s PA development.

Despite the importance of these three familial factors to early literacy skills, their combined contribution to early literacy was less than that of within-child factors. The strongest and most consistent within-child predictor was letter knowledge, which comes as no surprise as it is well-established that knowledge of letter names and sounds allows the child insight into the alphabetic principle and has a strong relationship to later reading and spelling abilities [6], [88]. The ability to recall sentences verbatim was found to be an important predictor of early literacy skills when measured in Preschool and beginning Kindergarten. The nature of the sentence recall task requires listening skills and children who have developed these early in Preschool and beginning Kindergarten may be advantaged, correlating with their literacy development later on. Conversely, PA measured in Kindergarten, but not in Preschool, contributed to early literacy abilities. A possible reason for this is that there is wide individual variation in preschoolers across the range of PA skills (e.g., syllables vs. onset-rimes vs. phonemes), but due to developmentally-based improvement, explicit training and exposure to print in Kindergarten, this variation could have decreased, making PA a more sensitive predictor in Kindergarten. That is, at this point only those children would be identified with poor PA who are constitutionally unable to take advantage of activities aimed at developing PA, whereas earlier, these children would not have been easily distinguished from others in Preschool who were not maturationally ready to develop in some aspects of PA. Other significant predictors come into play at the end of Kindergarten, such as oral language and RAN. However, the significance of oral language was only marginal and RAN was only measured at the end of Kindergarten because of a lack of standardised measures before the age of 5 years. So although within-child factors accounted for greater variance in early literacy skills here, it will be important in the future to target those skills that proved most sensitive at the various time points in Preschool and Kindergarten to achieve the most accurate prediction of children’s early literacy skills in Year 1.

This study sought to augment the previously demonstrated predictive power for early literacy outcomes afforded by within-child factors through the addition of familial factors and in this we succeeded: school SES, family history and parents’ PA account for significant variance over and above within-child factors measured at Preschool and Kindergarten. This serves to illustrate that it may be as important to consider family factors as within-child measures when assessing children for risk of literacy difficulties. Yet the usefulness of the combination of family and within-child factors depends on when within-child factors are measured. Our findings suggest that additional family risk factors are most useful when children are at Preschool (4 years old) and at the end of Kindergarten. The beginning of Kindergarten marks the first time PA becomes critical and suggests that some threshold level of PA may be emerging at this time which masks the influence of familial factors.

### Identifying Children with Poor Year 1 Literacy Abilities

Using within-child factors only, we found that prospective classification of children performing below and above average in Year 1 early literacy tasks using within-child risk factors improved over time. Overall prediction accuracy improved from 65.7% (Preschool) to 82.4% (beginning Kindergarten) and sensitivity jumped from 47.5% to 77.5% respectively. It is noteworthy that the best classification accuracy using *only* within-child risk factors was obtained at the beginning of Kindergarten with a screening measure that consisted of letter knowledge, PA and sentence recall.

The addition of family risk factors in combination with child factors did provide better prediction success overall. The rate of classification success for the preschoolers (4 year olds) improved, even though previously authors such as Scarborough [67] have presented evidence that it is difficult to reliably identify children who may be at risk at such an early age. As noted above, family factors did not add significantly to prediction success at the beginning of Kindergarten but classification using a combination of within-child and family risk factors was most accurate at the end of Kindergarten, with high levels of both sensitivity and specificity. This extends the findings of Schatschneider, Fletcher, Francis, Carlson and Foorman [6], who reported a stronger association between pre-literacy predictors measured at the end of Kindergarten and early literacy skills in Year 1. Our data further suggest that adding family risk factors increases both sensitivity and specificity (i.e., overall classification accuracy = .88), which is considerably stronger than the Heath et al. [65] result. Such refinements are critical if we are indeed attempting, as Torgesen [89] has suggested to “catch them before they fall” (p. 32).

### Theoretical Implications

Our findings generally lend further credence to models of literacy which reflect causes from a variety of domains, such as biological (e.g., genetic), cognitive and environmental, that interact to produce the behavioural phenotype of a child with literacy difficulties [54], [90]. Indeed, we have shown that a combination of family factors (i.e., a family history of language and literacy problems, parents’ PA and school SES) and the child’s cognitive processes (e.g., PA, RAN, oral language) and learned skills (i.e., letter knowledge), best predicts early literacy skills.

Many family and twin studies have demonstrated that reading disabilities have a heritable component: Children who have parents with reading difficulties are more likely to display reading problems than those in the general population [91]. But this study is unique as far as we know in measuring PA for the first time in the parents of the children being examined, although, as mentioned above, it remains to be clarified to what extent this association is mediated by genetic and/or cultural influences. In future studies, it would be of interest to clarify the nature of the association by training parental PA to discover whether such input could influence children’s outcomes. Unfortunately, the weight of evidence outside the present study is for shared genes contributing significantly greater variance to early literacy than shared environments [13], [59], [92] which suggests that training of PA in parents would not in fact influence children’s outcomes. Even though PSE was found to contribute to some extent to children’s Year 1 literacy outcomes, this influence was not significant, which was surprising in view of previous research suggesting that PSE is related to higher levels of parent involvement in educational activities [61], [93]. This finding might suggest that parents with higher PSE who are more involved in their children’s schooling do not necessarily produce better literacy outcomes for their children. Or it may simply be that the contribution of the PSE scale mostly involves variance shared with the three family factors that were significant predictors of literacy outcomes (i.e., school SES, family history and parents’ PA). It is also possible that the PSE scale failed to fully capture parents’ beliefs about their capacity to support their children’s literacy development. Indeed, Walker, Wilkins, Dallaire, Samdler and Hoover-Dempsey [93] raise the question of how difficult it can be to create objective scale items that reliably tap beliefs regarding children’s education across a wide range of parents. Finally, this scale could have been subject to the common problems faced by any self-report measure which asks the respondent to reflect on their own cognitions; for a brief review of the issues with self-report measures see Haeffel and Howard [94].

### Practical Implications

High-risk children (i.e., three or more family risk factors) do not reach the literacy levels of low-risk children even after a year of formal education. These children may acquire skills at the same rate as their low-risk classmates, but exposure to standard literacy education does not allow them to redress the deficit with which they began. This continuing lag will clearly be a barrier to their ongoing schooling: Previous research has shown that children who have difficulties in Kindergarten and Year 1 continue to struggle in literacy skills in Year 4 [95], [96]. Further, we know that these literacy problems become increasingly resistant even to evidence-based intervention after Year 3 unless this input can be individualised and intensive, which is costly in terms of time and educational resources [96]. Our results highlight the crucial question of how educators can mitigate the influence of familial risk factors and emphasise the need to identify at-risk children earlier to minimise the chances of them falling behind.

Therefore, early screening of young children for difficulties in pre-literacy skills is important as it allows for identification of children at risk of literacy failure and in need of targeted intervention. Our findings show that this screening can take place for children as young as four years of age. At that age, the best predictors are letter knowledge and sentence recall, both of which can be cost-effectively measured and can be administered by teachers or education assistants with special training. When supplemented with knowledge of the child’s family (i.e., school SES, family history) and their parents’ PA (which can be assessed in 10 minutes by a teacher or education assistant at their child’s school), the rate of identifying children who are already starting to fall behind improves significantly. This will enable schools to provide appropriate instruction to their weakest students from the very beginning of their education. Continued screening and progress monitoring in Kindergarten (5 year olds) should include an additional assessment of PA for the children, but even this is a brief 10 minute test that can be conducted by teachers or education assistants.

Some may question the likelihood that parents would generally agree to participate in the information gathering needed to facilitate the effective identification of children who might be in need of extra and well-targeted help in their developing literacy skills. This is a fair observation, and it is worthwhile noting that 16 parent respondents (i.e., 10 percent of the larger study sample; *N* = 162) did not participate in the initial phonological awareness testing session. Although this is acknowledged as a substantial number, only two of these sixteen explicitly expressed a desire not to be tested in spite of being happy for their child to participate. For the remainder who went untested, anecdotal evidence suggests that time management was the major reason for their failure to participate, given that the initial PA testing was included as part of a parent training program involving a considerable time commitment. It is possible that scheduling of PA testing by familiar school staff within a normal school routine, but at the parents’ convenience, could remove this stumbling block.

Our results are particularly timely in view of the significant proportion of students failing at literacy in school systems worldwide where the opacity of English orthography is acknowledged as a significant barrier to literacy success [97]. For example, survey data [98] showed that 52 per cent of Australians aged 15 to19 had literacy skills that were “insufficient to meet the demands of everyday life and work in the emerging knowledge-based economy (p. 5)”, and according to the US National Academy of Sciences report on older adolescents and adults with low reading abilities [99], more than 90 million American adults lack adequate literacy skills. Researchers [100] have continually emphasised the imperative of early identification of children at high risk for literacy failure and much work has been done in this area already; further, the results of at least five national inquiries in the recent past [101]–[105] have converged on what can be regarded as best practice in literacy instruction. However, as Seidenberg [106] points out, educators have been regrettably slow to enact what the research has shown. Factors contributing to this lack of action could include uncertainty about the efficacy of predicting literacy outcomes at an individual level [107], how early in children’s lives accurate screening is possible, and how this can be done most cost-effectively. Moreover, well-designed training studies are required to demonstrate for parents and educators alike what literacy problems can and cannot be addressed outside of the school classroom. For example, whether or not boosting preschool children’s PA through parent PA training is possible and/or useful. Similarly, although children’s letter knowledge is a good predictor of literacy outcomes, experimental training of children’s early letter knowledge has been shown not to advance later reading skill [3], [108], [109]; so training children identified as being at high risk according to the combined family and within-child factors addressed here could confirm the inference from the literature and our present data that the uptake of training in letter-knowledge would be poorer in high risk children.

Such investigations could provide valuable insights into how educators can more effectively address genetically-influenced learning disabilities. The screening measure proposed here, which consists of a combination of child and family factors, may be of particular use in this endeavour. It is innovative because it captures prediction power in Preschool children that as far as we know has not been accessed previously; and it is sensitive (i.e., able to effectively differentiate children who are beginning to fall behind and those who are not), efficient and easily administered. But the present results also strongly suggest that children at high risk will require better designed and delivered literacy instruction, supported by equally expert interventions when and if their literacy development falters, than would otherwise be the case. This in turn implies that appropriate resourcing (e.g., pre-service teacher training in evidence-based literacy instruction; classroom staffing levels especially in the early school years) should go “hand in hand” with accurate early identification.

## Supporting Information

Table S1 **Correlations between the early literacy measures.**
(PDF)

Table S2 **Correlations between vaiables from time 1 to time 4.**
(PDF)

Table S3 **Rotated factor matrix for the Parental PSE Scale (N = 135).**
(PDF)
