# Supplementary material for: A Spotlight on Preschool: The Influence of Family Factors on Children’s Early Literacy Skills
Source: PLoS One. 2014 Apr 21;9(4):e95255. doi: 10.1371/journal.pone.0095255 (PMC3994054; doi:10.1371/journal.pone.0095255)
Supplement: Table S1 — Correlations between the early literacy measures. (PDF) [file pone.0095255.s001.pdf]

Table S1: Correlations (Spearmans rho) between the early literacy measures

Variable	WRMT-R Word Identification at (T3)	TOWRE Sight Word Efficiency	TOWRE Phonemic Decoding	TOWRE Word Reading Efficiency	WRMT-R Word identification	QUIL Non-word spelling	WIAT-II Spelling
Literacy Composite	.70	.93	.90	.93	.93	.79	.80
WRMT-R Word Identification (T3)		.73	.62	.69	.71	.48	.53
TOWRE Sight Word Efficiency			.92	.98	.94	.60	.70
TOWRE Phonemic Decoding				.97	.88	.62	.65
TOWRE Word Reading Efficiency					.94	.62	.69
WRMT-R Word identification						.59	.71
QUIL Non-word spelling							.50

All Correlations are significant at the 0.01 level (2-tailed).

NB: All scores except the Literacy Composite are Standard Scores, All measures were taken at T4 unless stated, Literacy composite = (TOWRE Word Reading Efficiency + WRMT-R Word Identification + QUIL Non-word spelling + WIAT-II Spelling)/4, Spearmans rho was used due to the skewed nature of some of the variables
