# Supplementary material for: A Spotlight on Preschool: The Influence of Family Factors on Children’s Early Literacy Skills
Source: PLoS One. 2014 Apr 21;9(4):e95255. doi: 10.1371/journal.pone.0095255 (PMC3994054; doi:10.1371/journal.pone.0095255)
Supplement: Table S2 — Correlations between vaiables from time 1 to time 4. (PDF) [file pone.0095255.s002.pdf]

Table S2: Correlations (Spearman's rho) between pre-literacy vaiables at time 1 throught to time 4, external (risk) factors, the T4 Literacy Composite, summed number of risk factors present (yes/no) based on median splits, and risk based on having 3 or more factors.

Variable (range of catogorical variable).	PA at T2	PA at T3	PA at T4	LK at T1	LK at T2	LK at T3	Recalling Sentences at T1†	Recalling Sentences at T2†	Recalling Sentences at T3†	Recalling Sentences at T4†	Language at T1†	Language at T2†	Language at T3†	Language at T4†	RAN (Objects & Colours) at T3†	School SES (1-10)	Mother's Education (1-7)	Parent PA at T1†	Parent's PSE at T1	First degree relative (n/y)	**T4 literacy composite**	No. risk factors (0-5)	Risk (low/high)
Phonological Awareness at T1	.60**	.48**	.41**	.58**	.51**	.38**	.35**	.31**	.39**	.30**	.39**	.55**	.42**	.38**	.21*	.08	.27**	.16	-.01	-.27**	.38**	-.23*	-.34**
Phonological Awareness at T2		.59**	.55**	.43**	.51**	.35**	.29**	.37**	.43**	.38**	.49**	.53**	.50**	.46**	.34**	.22*	.22*	.24*	-.04	-.16	.53**	-.27**	-.31**
Phonological Awareness at T3			.47**	.33**	.50**	.41**	.22*	.26**	.34**	.38**	.28**	.40**	.37**	.54**	.32**	-.07	.18	.22*	.10	-.21*	.50**	-.29**	-.30**
Phonological Awareness at T4				.25*	.45**	.38**	.25*	.31**	.34**	.37**	.31**	.38**	.38**	.49**	.25*	.16	.17	.40**	.12	-.29**	.73**	-.45**	-.44**
Letter Knowledge at T1					.74**	.46**	.14	.19	.20*	.18	.29**	.44**	.41**	.21*	.31**	.13	.27**	.02	.08	-.32**	.46**	-.26**	-.31**
Letter Knowledge at T2						.58**	.08	.19	.21*	.23*	.33**	.45**	.49**	.33**	.32**	.11	.24*	.10	.11	-.27**	.62**	-.32**	-.31**
Letter Knowledge at T3							.16	.20*	.17	.20*	.21*	.32**	.36**	.32**	.40**	-.03	.09	.03	.12	-.21*	.55**	-.12	-.15
Recalling Sentences at T1†								.60**	.47**	.46**	.53**	.33**	.25*	.43**	.07	.12	.13	.13	.03	.01	.25*	-.14	-.21*
Recalling Sentences at T2†									.60**	.69**	.48**	.53**	.37**	.57**	.12	.14	.18	.22*	.03	-.11	.38**	-.25*	-.29**
Recalling Sentences at T3†										.74**	.48**	.50**	.43**	.56**	.18	.18	.18	.21*	.03	-.16	.35**	-.25*	-.30**
Recalling Sentences at T4†											.44**	.48**	.40**	.76**	.16	.05	.13	.20*	.04	-.17	.35**	-.23*	-.29**
Core Language at T1†												.60**	.54**	.41**	.06	.27**	.31**	.15	-.02	-.11	.34**	-.27**	-.25*
Core Language at T2†													.60**	.51**	.21*	.17	.28*	.19	-.02	-.17	.43**	-.26**	-.28**
Core Language at T3†														.41**	.11	.18	.29**	.24*	.08	-.37**	.44**	-.41**	-.34**
Core Language at T4†															.41**	-.15	.04	.23*	.01	-.18	.48**	-.17	-.22*
RAN Objects & Colours at T3 †																-.09	-.12	.03	.12	.12	.44**	-.02	-.05
School SES (1-10)																	.35**	.06	-.20*	-.01	.28**	-.40**	-.21*
Mother's Level of Education (1-7)																		.20*	.05	-.23*	.25*	-.61**	-.50**
Parent PA at T1†																			.03	-.33**	.35**	-.56**	-.45**
Parent's PSE at T1																				-.13	.06	-.35**	-.31**
Has a first degree family member (sibling,parent) who has experienced literacy difficulties (no/yes)																					-.31**	.59**	.66**
T4 literacy composite																						-.48**	-.45**
Total number of external risk factors (0-5)																							.84**

**. Correlation is significant at the 0.01 level (2-tailed),*. Correlation is significant at the 0.05 level (2-tailed), † = Standard Scores,

NB: Spearmans rho was used due to the skewed nature of some of the variables, T4 Literacy Composite = (TOWRE Word Reading Efficiency + WRMT Word Identification + QUIL Non-word spelling + WIAT Spelling)/4
