# Supplementary material for: A Spotlight on Preschool: The Influence of Family Factors on Children’s Early Literacy Skills
Source: PLoS One. 2014 Apr 21;9(4):e95255. doi: 10.1371/journal.pone.0095255 (PMC3994054; doi:10.1371/journal.pone.0095255)
Supplement: Table S3 — Rotated factor matrix for the Parental PSE Scale (N = 135). (PDF) [file pone.0095255.s003.pdf]

Table S3: Rotated (direct oblimin with Kaiser normalization) factor matrix for the Parental PSE Scale (N= 135).

Response Items	Rotated Factor Loadings	
	1	2
I know how to get my child to listen.	**0.8**	0.466
I am able to manage my child.	**0.793**	0.346
I am able to help my child cope when they have a problem.	**0.657**	0.519
I know how I can help my child when he/she is sad.	**0.652**	0.371
I know what to do when child is worried.	**0.621**	0.472
I know about how my child's language will develop.	0.353	**0.694**
I feel confident in developing my child's early language and literacy skills.	0.511	**0.685**
I feel confident to develop good problem solving skills in my child.	**0.65**	**0.677**
I know about how my child will learn at school.	0.322	**0.671**
I can help my child learn to read and write.	0.418	**0.665**
I feel ready to help my child with schoolwork when the time comes.	0.468	**0.506**
Eigenvalues	4.95	1.36
% of variance	45.01	12.35
Cronbach’s Alpha (for subscales items only)	0.82	0.85

NB: Factor loadings on items included in the two subscale calculations appear in bold.
